# Utilizing a *Drosophila* Larval Model of Synucleopathy to Explore Mushroom Extract Treatments for Parkinson’s Disease

**DOI:** 10.17912/micropub.biology.002349

**Published:** 2026-09-19

**Authors:** Sarah Perry, Frankie Davis, Vishva Patel, Rudy Alvarado, Mckinley Black, Jefferson Bretanys Desca, Mason Briggs, Jackson Buck, Jontasia Campbell, Emma Carter, Dakota Clark, Aleandrjo Collins, Caleb Curtis, Sydney Denker, Machi Gillard, Lexie Goldammer, Tristen Gorman, Cameron Grayson, Daisy Heller, Kindall Holmes Cerveny, Josearmando Izaguirre, Colton James, Kelbe Jeffries, Seth Johnson, Elisha Keeler, Phoenix Kincade, Eric Lambert, Daniela Malagon, Emily Marks, Joseph Mcgeshick, Sarah McKinney, Zoey Melendrez, Gabriella Mignano, Jackson Miller, Hannah Miller, Jesse Perales, Cheena Pun, Trent Rollet, Shekinah Ross, David Rourke, Christian Ryan, Corey Slinker, Zoe Spann-Mcdonald, Jasper Spurk, Madelyn Steele, Emme Sylvester, Sarah Turpen, Tahmar Upshaw

**Affiliations:** 1 Austin Peay State University, Clarksville, TN, United States

## Abstract

Parkinson’s disease (PD) is characterized by dopaminergic neuron loss and progressive motor dysfunction. Here, we incorporated a *Drosophila* larval model of α-synucleinopathy into a course-based undergraduate research experience (CURE) to investigate the effects of mushroom extract supplementation on locomotor dysfunction. Larvae expressing human A53T α-synuclein in dopaminergic neurons exhibited impaired Ring Maze performance. Dietary supplementation with Lion’s mane ( *Hericium erinaceus* ) improved overall Ring Maze performance in A53T larvae; however, Lion’s mane also enhanced performance in TH-Gal4 controls, suggesting a broader locomotor-enhancing effect rather than an A53T-specific rescue. In contrast, vitamin C improved performance in A53T larvae without a detectable beneficial effect in controls. Turkey tail ( *Trametes versicolor* ) and Chaga ( *Inonotus obliquus* ) did not consistently improve A53T performance. These findings identify Lion’s mane as an interesting candidate for further study while demonstrating the utility of the Ring Maze for undergraduate investigation of neurodegenerative disease models and treatment effects.

**Figure 1. Lion’s mane improves Ring Maze performance in control and A53T α-synuclein-expressing larvae:** A) Schematic depicting the Ring Maze. A single wandering third-instar larva is placed in the center of a 100-mm 1% agarose plate positioned over a template containing a 60-mm-diameter ring. The time required for the larva to reach the edge of the ring is recorded for up to 60 s. B) Ring Maze failure rates for control (TH-Gal4/+) and α-synuclein larvae (TH-Gal4 > UAS-αSyn.A53T) with and without supplement treatments. Failure rate is a categorical measure of task completion and was compared using chi-squared tests (N = 60–145). C) Time to edge for successful larvae only. This continuous measure describes performance among animals that completed the task within 60 s; individual observations and mean ± SEM are shown. Groups were compared using two-tailed Student’s t-tests (N = 44–122). D–E) Cumulative frequency distributions showing the proportion of larvae reaching the edge over time for TH-Gal4 (D) and A53T (E) groups. Failed trials were assigned a value of 61 s so that both completion rate and completion time are represented, making these plots the most comprehensive representation of overall Ring Maze performance across the full experimental population. Pairwise comparisons with the corresponding untreated group were performed using Kolmogorov–Smirnov tests (N = 60–145). (ns) p > 0.05, *p < 0.05, **p < 0.01, ***p < 0.001. ****p < 0.0001

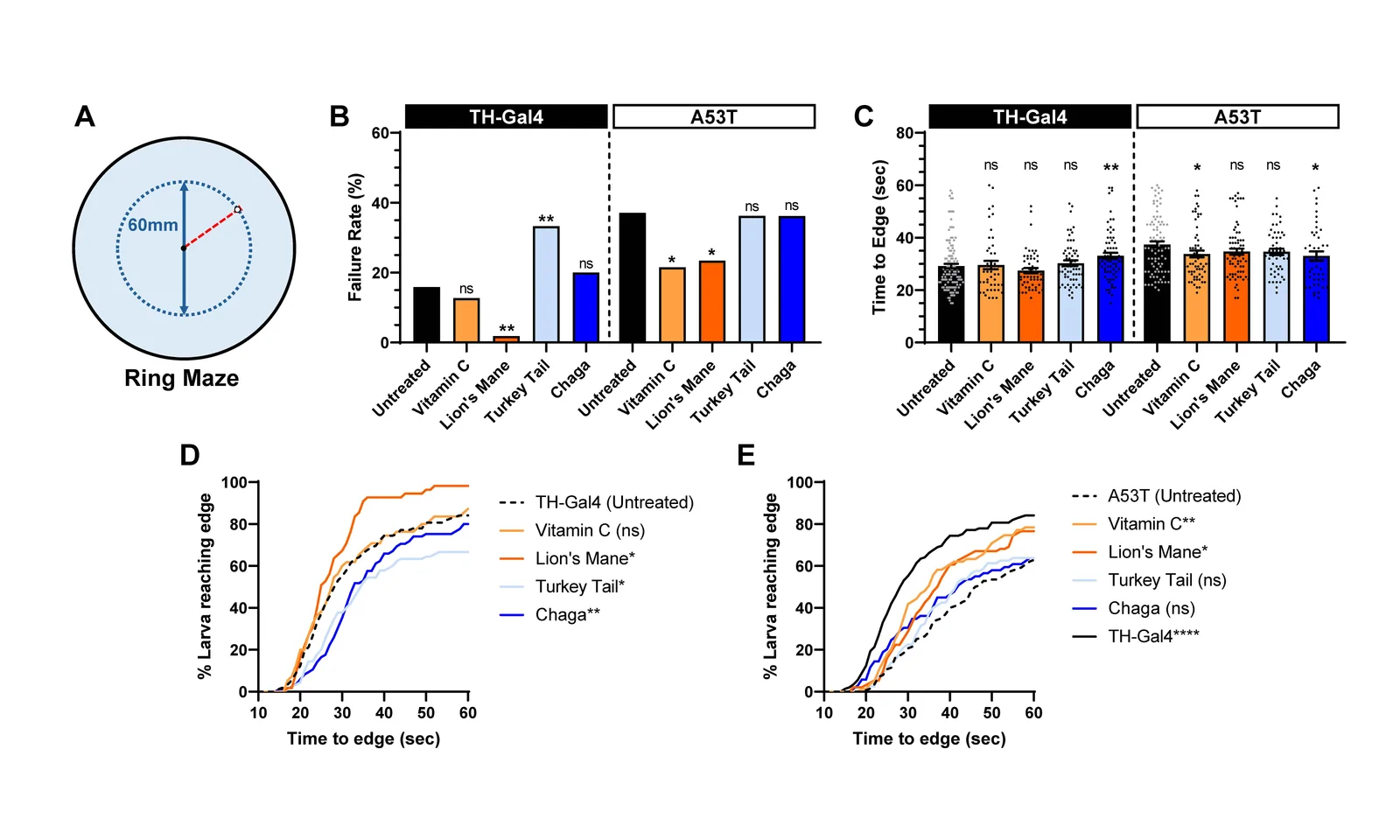



## Description

Parkinson’s disease (PD) is a common neurodegenerative disorder characterized by the progressive loss of dopaminergic neurons and resulting motor dysfunction (Aryal & Lee, 2019; Suzuki et al., 2022). Although treatments such as L-Dopa provide symptomatic relief, they are associated with significant long-term side effects, highlighting the need for alternative therapeutic strategies (Blosser et al., 2020). The genetic and environmental complexity of PD presents challenges for traditional model systems; however, *Drosophila melanogaster* larvae offer a powerful and tractable model for studying disease mechanisms (Blosser et al., 2020; Perry et al., 2026; Perry & More, 2025; Varga et al., 2014).

Expression of human α-synuclein variants in *Drosophila* can recapitulate PD-associated phenotypes, including locomotor dysfunction. In our previous systematic comparison of wild-type human α-synuclein and five disease-associated variants (A30P, E46K, H50Q, G51D, and A53T), dopaminergic neurons showed pronounced vulnerability to α-synuclein, particularly the E46K and A53T variants (Perry & More, 2025). A53T has also been used as a representative genetic PD model in *Drosophila* larvae, where dopaminergic expression produces locomotor deficits and dopaminergic neuron loss and is responsive to pharmacological and environmental manipulation (Varga et al., 2014; Blosser et al., 2020). We therefore selected TH-Gal4 > UAS-αSyn.A53T as a previously validated PD genotype for the present supplement screen rather than repeating the α-synuclein variant comparison.

Mushroom-derived supplements, including Lion’s mane, Turkey tail, and Chaga, contain bioactive compounds with reported neuroprotective properties, yet their effects in neurodegenerative contexts remain underexplored (Godela et al., 2025). Here, we leverage the *Drosophila* larval synucleopathy model within a course-based undergraduate research experience (CURE) to evaluate whether these supplements modify locomotor performance in A53T-expressing larvae.

To examine locomotor phenotypes, we employed a simple behavioral assay we refer to as the “Ring Maze,” which assesses a larva’s ability to navigate from the center of an arena to a defined edge ( Figure 1A ). This assay is low-tech and straightforward for students to learn, making it well suited for a CURE project. We previously used the Ring Maze to examine antioxidant supplementation in α-synuclein-expressing larvae (Perry et al., 2026).

An additional educational advantage of the Ring Maze is that a single behavioral dataset can be analyzed using multiple complementary statistical approaches. Students first evaluate task completion as a categorical variable by comparing success and failure frequencies using a chi-squared test. Among larvae that successfully complete the task, time to edge provides a continuous quantitative variable that can be compared using tests appropriate for continuous data. Finally, cumulative frequency distributions incorporate both time to edge and unsuccessful trials by assigning failed larvae a value of 61 s. Because this analysis retains both successful and failed trials, the cumulative distribution provides the most comprehensive representation of overall Ring Maze performance across the full experimental population. Together, these analyses allow students to examine complementary components of the same behavioral phenotype while gaining experience selecting statistical approaches appropriate for different types of variables.

Larval locomotion was assessed across control (TH-Gal4) and PD model (A53T) genotypes under different dietary conditions. As expected, A53T-expressing larvae exhibited impaired Ring Maze performance relative to TH-Gal4 controls, including an increased failure rate ( Figure 1B, p = 7.93 × 10⁻⁶) and increased time to edge among successful larvae ( Figure 1C, p < 0.0001).

The separate analyses revealed that time to edge among successful larvae was relatively similar across several treatment groups despite differences in failure rate and cumulative performance. This distinction reflects the conditional nature of the successful-trial analysis: larvae that do not complete the task within 60 s are excluded from Figure 1C . Thus, groups can contain successful larvae with similar completion times while differing substantially in the proportion of the total population capable of completing the task. The cumulative frequency distributions ( Figure 1D –E), which include failed trials, therefore provide the most holistic assessment of overall population performance.

In TH-Gal4 control larvae, Lion’s mane improved Ring Maze performance, whereas Turkey tail and Chaga impaired performance and vitamin C had no detectable beneficial effect ( Figure 1B –D). In A53T larvae, both Lion’s mane and vitamin C improved overall Ring Maze performance compared with untreated A53T larvae, whereas Turkey tail and Chaga did not improve performance ( Figure 1B –E). Importantly, the beneficial effect of Lion’s mane in both TH-Gal4 and A53T larvae indicates that the present data do not establish an A53T-specific rescue. Rather, Lion’s mane appears to exert a broader locomotor-enhancing effect that is sufficient to improve performance in larvae expressing pathogenic A53T α-synuclein. Vitamin C showed a different pattern, improving A53T performance without a detectable beneficial effect in TH-Gal4 controls.

Lion’s mane therefore emerges as an intriguing candidate for further investigation, although the interpretation of its effect should remain cautious. Previous work in diverse experimental systems has reported neuroprotective or neurotrophic effects of Lion’s mane and its constituents (Brandalise et al., 2023; Cordaro et al., 2022; Tripodi et al., 2022). Lion’s mane ( *Hericium erinaceus* ) contains bioactive compounds including hericenones, erinacines, polysaccharides, and antioxidant molecules. Ergothioneine, a dietary antioxidant found in mushrooms, has demonstrated protective effects in a *Caenorhabditis elegans* model of PD (Gao et al., 2025), and Lion’s mane extracts have been reported to reduce α-synuclein aggregation in cell culture systems (Tripodi et al., 2022). These observations provide plausible avenues for future mechanistic study.

However, the present behavioral experiments cannot distinguish among direct neuroprotection, modification of α-synuclein-associated pathology, or a more general effect on larval physiology or locomotor behavior. In addition, mushroom extracts were examined at a single concentration (50 mg/mL), so this study does not establish dose dependence or determine an optimal treatment concentration. Future experiments examining multiple concentrations and mechanistic endpoints will be needed to resolve these possibilities. Nevertheless, the improvement in overall Ring Maze performance following Lion’s mane supplementation supports continued investigation of its effects in *Drosophila* models of synucleinopathy.

## Methods

**Fly genetics and husbandry:**

Flies were reared under standard conditions on cornmeal food (NutriFly, Bloomington formulation) at 25°C in a 12-hour light/dark cycle incubator. Larval density for crosses was controlled by pairing 6 females with 3–5 males and allowing the crosses to seed for 2–3 days before transferring the parents to a new tube. Wandering third-instar larvae were typically observed on day 5 or 6 of culturing. Control genotypes were generated by outcrossing TH-Gal4 females to w1118 males, and the resulting progeny were used for experiments. The TH-Gal4/+ group therefore provides the genetic baseline for assessing the effect of A53T expression as well as treatment effects in the absence of the UAS-A53T transgene. A wild-type human α-synuclein misexpression group was not repeated in this treatment screen because wild-type and mutant α-synuclein variants had been systematically compared in our previous optimization study, which identified A53T as a robust dopaminergic locomotor phenotype for subsequent intervention studies (Perry & More, 2025). Fly stocks used in this study (obtained from the Bloomington Drosophila Stock Center, BDSC) were as follows: w1118 (Perry lab stock), TH-Gal4 (BDSC_8848), and UAS-A53T (BDSC_8148).

**Supplement treatments:**

Mushroom powder supplements (Lion’s mane, Turkey tail, and Chaga) were acquired from BulkSupplements and added to molten fly food at a concentration of 50 mg/mL. This concentration was used as a single-dose exploratory screen across mushroom supplements; a formal dose-response analysis was outside the scope of the present CURE study. Cordyceps powder (BulkSupplements, 50 mg/mL) was also examined; however, larvae were not easily cultured on this medium. We plan to examine this effect in more depth in future work. Vitamin C (0.25 mg/mL) was used as a positive control because it has been shown to improve α-synuclein-related motor deficits in previous studies (Perry et al., 2026; Perry & More, 2025).

**Behavioral Assays:**

The Ring Maze is described in detail in our previously published study (Perry et al., 2026), and a detailed, user-friendly protocol is attached as supplement. Individual wandering third-instar larvae were collected with a damp brush, rinsed briefly in water to remove residual food, and transferred to the center of a fresh 100-mm plate containing 1% agarose positioned over a template containing a 60-mm-diameter ring. Larvae were allowed to acclimate and reorient, and timing began once the larva initiated movement away from the center of the arena. The trial ended when the larva’s mouth hooks reached the edge of the 60-mm ring or after a maximum of 60 s. Larvae reaching the ring within 60 s were scored as successful, and elapsed time was recorded. Larvae that did not reach the ring within 60 s were scored as failures. Failure rate was analyzed as a categorical outcome using chi-squared tests. Time to edge was analyzed as a continuous outcome among successful trials using two-tailed Student’s t-tests. For cumulative frequency distributions, failed larvae were assigned a value of 61 s so that all animals contributed to the population-level analysis; distributions were compared using Kolmogorov–Smirnov tests. A fresh agarose plate was used for each trial.
